# Association of Propofol Versus Midazolam Sedation With Successful Pharyngeal Observation During Upper Gastrointestinal Endoscopy: A Retrospective Study

**DOI:** 10.1002/deo2.70422

**Published:** 2026-09-03

**Authors:** Kazuya Sumi, Tomoya Sato, Tomona Sakurai, Kota Uehara, Akira Ishihara, Takayoshi Ito

**Affiliations:** ^1^ Digestive Diseases Center Showa Medical University Koto‐Toyosu Hospital Tokyo Japan; ^2^ Hitachi Medical Center Hospital Ibaraki Japan; ^3^ Division of Gastroenterology Showa Medical University School of Medicine Tokyo Japan

**Keywords:** esophagogastroduodenoscopy, midazolam, pharyngeal observation, propofol, sedation

## Abstract

**Objectives:**

Adequate pharyngeal observation during esophagogastroduodenoscopy (EGD) is increasingly recognized as an important component of high‐quality upper gastrointestinal endoscopy. However, visualization may be impaired by gag reflexes and patient movement. The current study evaluated the association between sedation strategy and successful pharyngeal observation during routine EGD. Further, whether this association differs according to operator experience was examined.

**Methods:**

This retrospective single‐center observational study included consecutive sedated EGD procedures performed between April 2024 and March 2025. Successful pharyngeal observation was defined as the successful identification of predefined pharyngeal and hypopharyngeal anatomical landmarks. Multivariate logistic regression analysis was performed to identify factors independently associated with successful pharyngeal observation. Interaction, sensitivity, and propensity score‐matched analyses were also conducted.

**Results:**

In total, 444 EGD procedures, including 245 performed under midazolam sedation and 199 under propofol sedation, were analyzed. The propofol group had a significantly higher successful pharyngeal observation rate than the midazolam group (90.0% vs. 79.2%, *p* = 0.011). In the multivariate analysis, propofol sedation was independently associated with successful pharyngeal observation (odds ratio: 2.18, 95% confidence interval: 1.20–3.96, *p* = 0.008). Sensitivity analyses excluding biopsy cases and analyses restricted to trainees yielded consistent results. Propensity score‐matched analysis also yielded consistent results.

**Conclusions:**

Sedation strategy was independently associated with successful pharyngeal observation during routine EGD. Propofol sedation may facilitate a more consistent pharyngeal visualization across operators with varying levels of experience.

**Trial Registration:**

N/A.

**Abbreviations**
ASA‐PS: American Society of Anesthesiologists physical status
BMI: body mass index
CI: confidence interval
EGD: esophagogastroduodenoscopy
JGES: Japan Gastroenterological Endoscopy Society
MOAA/S: Modified Observer's Assessment of Alertness/Sedation
OR: odds ratio.

## 1 Introduction

Esophagogastroduodenoscopy (EGD) is one of the most frequently performed endoscopic procedures worldwide, and it represents a foundational procedure in endoscopic training. Ensuring high‐quality mucosal inspection while maintaining patient safety is important to contemporary endoscopic practice. Recent international quality initiatives have increasingly emphasized standardized mucosal visualization, photodocumentation, adequate inspection time, and procedural quality indicators in upper gastrointestinal endoscopy [1, 2].

Standardized quality indicators for pharyngeal observation have not yet been established. However, cautious inspection of the pharyngeal and hypopharyngeal regions during EGD has increasingly been recognized as clinically important for early lesion detection and comprehensive upper gastrointestinal examination [3, 4]. Adequate pharyngeal visualization remains technically challenging because gag reflexes, swallowing reflexes, and patient movement may interfere with stable visualization [5, 6]. Previous feasibility studies have shown that laryngeal and hypopharyngeal visualization can be incorporated into routine EGD, although gagging and inadequate sedation may result in incomplete visualization [7].

Sedation during EGD has traditionally been discussed primarily in terms of patient comfort and procedural safety. However, appropriate sedation may also contribute to improved procedural conditions and examination quality for both patients and endoscopists during gastrointestinal endoscopy [8, 9, 10]. Further, the guidelines for sedation in gastroenterological endoscopy issued by the Japan Gastroenterological Endoscopy Society (JGES) emphasize that sedation improves patient receptivity and enhances overall examination and treatment performance [9]. In particular, a multicenter prospective trial revealed that propofol sedation facilitates a more complete pharyngeal examination and significantly improves the detection rate of small superficial squamous cell carcinomas in high‐risk patients [11]. Sedation may therefore improve examination quality by suppressing gag reflexes, facilitating stable visualization, and potentially improving lesion detection [12, 13, 14].

Previous studies comparing midazolam and propofol have focused mainly on safety, patient satisfaction, and procedural tolerance, with limited data on procedural quality, particularly pharyngeal observation [10, 12, 14].

Procedural conditions during EGD may vary according to operator experience and sedation practice, particularly in regional training hospitals where endoscopists with various training backgrounds perform examinations in real‐world settings. Therefore, this study aimed to evaluate the association between sedation strategy and successful pharyngeal observation during routine EGD and to determine whether this association differs according to operator experience.

## 2 Methods

### 2.1 Study Design

This retrospective, single‐center observational study was conducted at Hitachi Medical Center Hospital, a regional teaching hospital in Japan where trainees from multiple institutions rotate for endoscopic training. Consecutive EGDs performed under intravenous sedation between April 2024 and March 2025 were reviewed. No formal a priori sample size calculation was performed because of the retrospective observational design. The one‐year study period was predefined to encompass the institutional trainee rotation cycle, and all eligible examinations performed during this period were included. The institutional review board approved the study protocol (approval number: 251111‐1).

### 2.2 Indication Classification

The indications for EGD were categorized as screening or follow‐up. The screening indications included examinations performed for screening purposes or diagnostic evaluation of abnormalities detected during routine health checkups. The follow‐up category included surveillance of previously identified benign or neoplastic conditions and post‐treatment surveillance. Patients with American Society of Anesthesiologists physical status (ASA‐PS) I–III were eligible. Both screening and follow‐up outpatient examinations were included in the analysis. Biopsy was performed when clinically indicated for suspicious mucosal abnormalities in the esophagus, stomach, or duodenum. No biopsies were performed for pharyngeal lesions.

### 2.3 Operators

Four endoscopists (one board‐certified supervising endoscopist with extensive experience [greater than several thousand EGDs carried out under both midazolam and propofol sedation] and three trainees) performed EGD procedures during the study period.

All trainees had completed 2 years of general residency training, followed by 1 year of specialty training at their respective endoscopy training institutions before rotating to our hospital. Therefore, at the time of this study, they were in their second or third year of endoscopy training. Each trainee had performed >200 upper gastrointestinal endoscopy procedures before the rotation. The sedation training backgrounds of the trainees differed. In particular, one trainee had primarily been trained using midazolam‐based sedation, whereas two trainees had previous experience with propofol‐based sedation. At our institution, the trainees received structured instruction in propofol administration under direct supervision. In addition, they were instructed at the beginning of their rotation to routinely perform pharyngeal observation during EGD, including visualization of predefined pharyngeal and hypopharyngeal anatomical landmarks.

### 2.4 Sedation Protocol

Sedation type (propofol or midazolam) was determined according to the availability of institutional staffing and with reference to the Japan Gastroenterological Endoscopy Society sedation guidelines [9]. Propofol sedation was performed only on days when at least two physicians were available to ensure adequate monitoring and procedural support. Otherwise, midazolam was used. Accordingly, the sedative agent was determined primarily by daily staffing conditions rather than patient characteristics or anticipated procedural difficulty.

EGD procedures were performed using standard high‐definition endoscopes (XZ1200 or HQ290; Olympus Medical Systems, Tokyo, Japan). All patients received topical pharyngeal anesthesia with lidocaine spray prior to the procedure. Patients received five squirts of lidocaine 10% spray. Sedation was performed using either propofol or midazolam alone. No opioid analgesics, including pethidine or pentazocine, were administered in either sedation group, and the Valsalva maneuver or vocalization was not routinely used during pharyngeal observation.

Propofol sedation was initiated at a dose of 0.5–1.0 mg/kg intravenously for induction. Additional boluses of 10–20 mg were administered as needed in response to patient movement or insufficient sedation until adequate sedation was achieved. Sedation depth was clinically targeted to moderate‐to‐deep sedation. Formal Modified Observer's Assessment of Alertness/Sedation (MOAA/S) scores were not prospectively recorded. However, the intended sedation level generally corresponded to a MOAA/S score of approximately 2–3 [15].

Midazolam was administered intravenously at an initial dose of 0.05–0.07 mg/kg. Additional 1‐mg increments were provided as needed according to patient response. Similarly, sedation depth was targeted to moderate‐to‐deep sedation.

### 2.5 Monitoring and Discharge Criteria

All patients were continuously monitored using electrocardiography and pulse oximetry. Noninvasive blood pressure was measured at 5‐min intervals throughout the procedure.

Oxygen desaturation was defined as an SpO_2_ of <90%, and hypotension was defined as a systolic blood pressure of <90 mmHg or a ≥ 20% decrease in systolic blood pressure from baseline.

Patients were discharged when their modified Aldrete score reached ≥9.

### 2.6 Outcomes

The primary outcome was successful pharyngeal observation.

**FIGURE 1:**
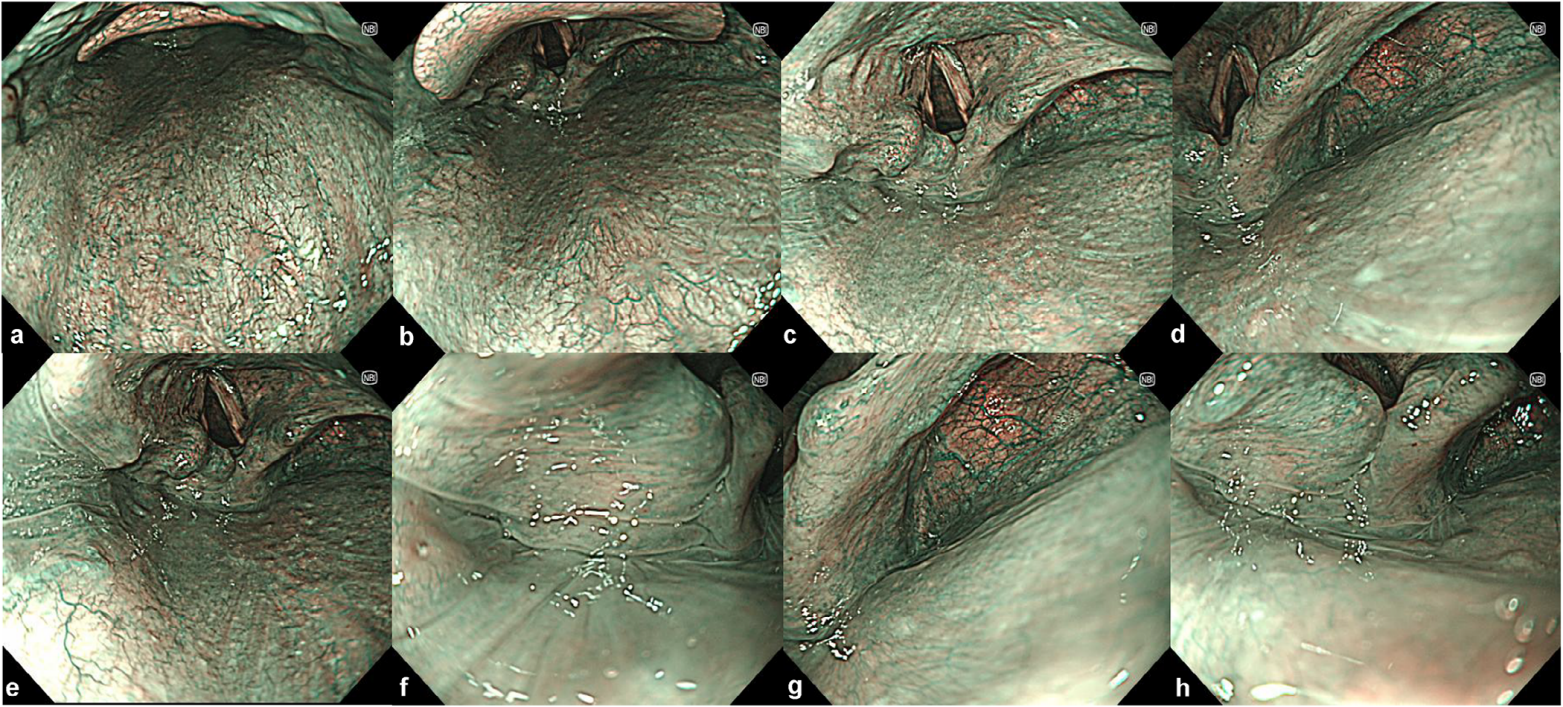
Representative endoscopic images of the predefined pharyngeal and hypopharyngeal anatomical landmarks used to assess successful pharyngeal observation. (a) Posterior pharyngeal wall. (b) Epiglottis. (c) Arytenoid region and vocal folds. (d) Right lateral pharyngeal wall. (e) Left lateral pharyngeal wall. (f) Left pyriform sinus. (g) Right pyriform sinus. (h) Interarytenoid region.

Successful pharyngeal observation was defined as the successful identification of all predefined pharyngeal and hypopharyngeal anatomical landmarks (the epiglottis, arytenoid region, vocal folds, posterior pharyngeal wall, lateral pharyngeal wall, and pyriform sinus) during insertion or withdrawal, as documented on recorded images (Figure 1). These landmarks were selected to represent clinically relevant pharyngeal and hypopharyngeal structures routinely assessed during pharyngeal examination.

All recorded images were independently reviewed twice by the same supervising endoscopist on separate occasions in a blinded manner. Cases were classified as successful only when both evaluations confirmed adequate visualization. If inadequate visualization was identified in either assessment, the case was classified as unsuccessful.

The secondary outcomes included biopsy performance, oxygen desaturation, hypotension, patient satisfaction, and patient discomfort scores. After recovery, patient satisfaction and discomfort were assessed using a 10‐point Visual Analog Scale.

Procedure time was evaluated as an exploratory procedural metric to assess whether the sedation method was associated with overall procedural efficiency.

### 2.7 Statistical Analysis

Continuous variables were expressed as the mean ± standard deviation and compared using the Student's *t*‐test. Categorical variables were compared using the chi‐square test or the Fisher's exact test as appropriate.

Multivariate logistic regression analysis was performed to identify independent factors associated with successful pharyngeal observation. The covariates included sedation type, operator status, age, sex, body mass index (BMI), ASA classification, biopsy performance, calendar period, and indication (screening vs. follow‐up). The one‐year study period was divided into four consecutive 3‐month periods (P1–P4) to account for potential temporal changes in trainee experience and procedural learning. Covariates were selected based on their potential clinical relevance to the primary outcome and observed differences in baseline or procedural characteristics between the sedation groups.

To assess whether the association between sedation type and successful pharyngeal observation differed according to operator experience, an interaction term between sedation type and operator status was included in a separate multivariable model using the same covariates. Sensitivity analyses excluding biopsy cases and restricting the analysis to trainee‐performed procedures were conducted to assess the robustness of the primary findings. In addition, propensity score matching was performed to reduce potential confounding between the sedation groups. Propensity scores were estimated using calendar period, age, sex, BMI, ASA‐PS, operator experience, examination indication, and history of previous endoscopy. One‐to‐one nearest‐neighbor matching without replacement was performed using a caliper of 0.2 of the standard deviation of the logit of the propensity score. Covariate balance after matching was assessed using standardized differences. Outcomes were compared between the matched groups using analyses accounting for the matched‐pair structure.

As an exploratory analysis, linear regression analysis was performed to identify factors independently associated with the procedure time, including sedation type, operator status, biopsy performance, calendar period, and indication.

## 3 Results

### 3.1 Study Population

**FIGURE 2:**
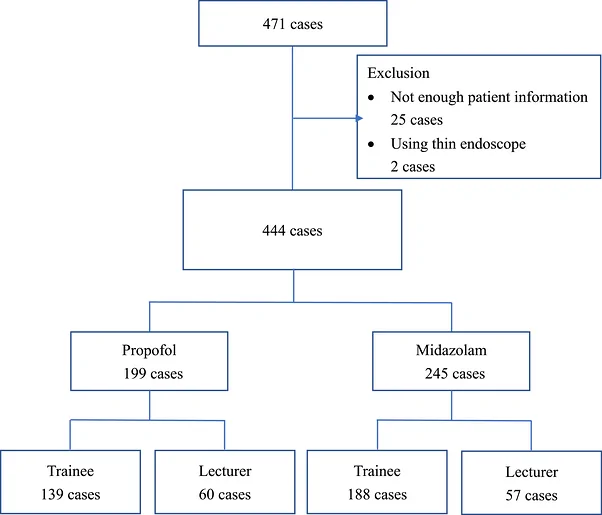
Flow diagram of the patient selection process.

In total, 471 sedated EGD procedures were screened during the study period. The study flow and patient selection process are shown in Figure 2. After the exclusion of 27 cases due to insufficient clinical data or use of transnasal endoscopy, 444 procedures were included in the final analysis. Of the 444 procedures that were included, 327 were performed by trainees (*n* = 139 in the propofol group and *n* = 188 in the midazolam group), and 117 were conducted by instructors (*n* = 60 and 57, respectively).

Among these, 245 procedures were carried out under midazolam sedation and 199 under propofol sedation. The baseline characteristics of the patients were comparable between the two groups (Table 1).

**TABLE 1:** Characteristics of the patients according to sedation agent (*n* = 444).

	Overall (*n* = 444)	Propofol group (*n* = 199)	Midazolam group (*n* = 245)	*p*‐Value
Age, years (mean ± SD)		65.5 ± 16.2	67.9 ± 15.3	0.1112
Sex, male (%)	53.6% (238)	50.8% (101/199)	55.9% (137/245)	0.3381
BMI, kg/m^2^; (mean ± SD)		23.4 ± 4.2	23.2 ± 4.0	0.6889
ASA‐PS I, II	97.7% (434)	97.5% (194/199)	98.0% (240/245)	0.7584
ASA‐PS III	2.3% (10)	2.5% (5/199)	2.0% (5/245)
Respiratory disease (%)	8.8% (39/444)	7.5% (15/199)	9.8% (24/245)	0.5006
Cardiovascular disease (%)	14.2% (63/444)	13.6% (27/199)	14.7% (36/245)	0.7854
Liver disease (%)	2.5% (11/444)	3.0% (6/199)	2.0% (5/245)	0.5520
Renal disease (%)	1.1% (5/444)	0.5% (1/199)	1.6% (4/245)	0.3856
Intracranial disease (%)	5.6% (25/444)	5.5% (11/199)	5.7% (14/245)	1.0000
Psychiatric disorder (%)	3.8% (17/444)	2.5% (5/199)	4.9% (12/245)	0.2220
Use of antithrombotic agents (%)	19.1% (85/444)	16.6% (33/199)	21.4% (52/245)	0.2258
First‐time endoscopy (%)	19.6% (87/444)	20.6% (41/199)	18.8% (46/245)	0.6329
Screening (%)	77.8% (346/444)	74.4% (148/199)	80.8% (198/245)	0.1086
Trainee	73.6% (327/444)	69.9% (139/199)	76.7% (188/245)	0.1055
**Period**				<0.0001
Period 1	15.5% (69/444)	13.1% (26/199)	17.6% (43/245)	
Period 2	32.7% (145/444)	30.7% (61/199)	34.2% (84/245)	
Period 3	24.5% (109/444)	18.6% (37/199)	29.4% (72/245)	
Period 4	27.3% (121/444)	37.6% (75/199)	18.8% (46/245)	
Mean dose, mg (mean ± SD)		63.9 ± 22.0	3.6 ± 0.9	

Abbreviations: ASA‐PS, American Society of Anesthesiologists physical status; BMI, body mass index.

### 3.2 Overall Endoscopic Outcomes

The propofol group had a significantly higher successful pharyngeal observation rate than the midazolam group (90.0% [179/199] vs. 79.2% [194/245], *p* = 0.0110).

Biopsy was performed less frequently in the propofol group than in the midazolam group (10.6% vs. 18.8%, *p* = 0.0167). The rates of hypotension, oxygen desaturation, patient satisfaction, and patient discomfort did not significantly differ between the two groups (Table 2).

**TABLE 2:** Comparison of endoscopic outcomes between sedation types in all procedures.

	Propofol group (*n* = 199)	Midazolam group (*n* = 245)	*p*‐Value
Successful pharyngeal observation (%)	90.0 % (179/199)	79.2% (194/245)	0.0110
Procedure time, min (mean ± SD)	6.7 ± 2.4	7.9 ± 3.0	<0.0001
Biopsy performed (%)	10.6% (21/199)	18.8% (46/245)	0.0167
Hypotension (%)	12.6% (25/199)	12.7% (31/245)	1.0000
Oxygen desaturation (%)	13.6% (27/199)	8.6% (21/245)	0.1237
Patient satisfaction (0–10)	9.6 ± 1.3	9.5 ± 1.5	0.5558
Patient discomfort	0.6 ± 1.7	0.7 ± 1.9	0.5533

Abbreviation: VAS, Visual Analog Scale.

The propofol group had a shorter procedure time than the midazolam group (6.7 ± 2.4 vs. 7.9 ± 3.0 min, *p* < 0.0001). However, procedure duration may be influenced by multiple procedural factors. Thus, it was considered a downstream procedural variable and was not included in the primary multivariate model.

### 3.3 Multivariate Analysis for Successful Pharyngeal Observation

In multivariate analysis (Table 3), propofol use was independently associated with a higher likelihood of successful pharyngeal observation (odds ratio [OR]: 2.18, 95% confidence interval [CI]: 1.20–3.96, *p* = 0.008). Operator experience was also an independent factor, with supervising endoscopists achieving higher success rates than trainees (OR: 3.11, 95% CI: 1.41–6.84, *p* = 0.002). Older age was also independently associated with successful pharyngeal observation (OR: 1.02 per year, 95% CI: 1.00–1.04, *p* = 0.028). Other covariates were not significantly associated with successful pharyngeal observation.

**TABLE 3:** Multivariate logistic regression analysis for successful pharyngeal observation.

Variables	Odds ratio	95% CI	*p*‐Value
Propofol sedation	2.18	1.20–3.96	0.008
Operator (expert vs. trainee)	3.11	1.41–6.84	0.002
Examination period	—	—	0.257
ASA (III vs. I–II)	0.36	0.08–1.64	0.210
Indication (follow‐up vs. screening)	1.27	0.62–2.60	0.501
Biopsy	0.99	0.48–2.04	0.983
Age	1.02	1.00–1.04	0.028
Sex (M vs. F)	0.84	0.49–1.46	0.550
BMI	0.97	0.91–1.04	0.370

Abbreviations: ASA‐PS, American Society of Anesthesiologists physical status; BMI, body mass index.

### 3.4 Interaction Analysis

The interaction between propofol use and operator experience was not significant (*p* = 0.787), suggesting that the association between propofol sedation and successful pharyngeal observation did not significantly differ according to operator experience.

### 3.5 Sensitivity Analyses

In a sensitivity analysis excluding procedures with biopsy (*n* = 377), propofol sedation remained independently associated with successful pharyngeal observation (OR: 2.46, 95% CI: 1.29–4.68, *p* = 0.005; Table S1).

In the analysis restricted to trainees (*n* = 327), propofol sedation was also independently associated with successful pharyngeal observation (OR: 2.39, 95% CI: 1.23–4.63, *p* = 0.008; Table S2).

Propensity score matching generated 166 matched pairs (332 patients). Baseline characteristics of the matched cohort are presented in Table S3. Covariate balance was substantially improved after matching. Successful pharyngeal observation remained significantly more frequent in the propofol group than in the midazolam group (90.1% [150/166] vs. 80.7% [134/166], *p* = 0.0136) (Table S4).

### 3.6 Exploratory Analysis of Procedure Time

In the multivariate analysis, procedure time was associated with several procedural factors, including sedation type, biopsy performance, operator experience, and examination period (Table S5).

## 4 Discussion

This real‐world study showed that propofol sedation was associated with successful pharyngeal observation during upper gastrointestinal endoscopy. In particular, propofol sedation was associated with a significantly higher rate of successful pharyngeal observation.

This association remained significant in the multivariable analysis and was consistently supported by sensitivity analyses, including the trainee‐restricted analysis, as well as by propensity score matching, suggesting that the finding was robust to adjustment for potential confounding and differences in operator experience. Previous studies have reported that sedation is associated with improved detection of small upper gastrointestinal neoplasms [16]. Lesion detection depends on adequate mucosal visualization. Thus, successful pharyngeal observation may represent an important component of examination quality during routine EGD.

EGD is a fundamental procedure in endoscopic training, and achieving stable visualization during the insertion phase can be an important early technical milestone for trainees. Adequate visualization of the pharynx during EGD can be technically challenging, particularly for less experienced endoscopists. The insertion phase of EGD frequently provokes the gag reflex and patient movement, which may interfere with cautious inspection of pharyngeal structures [12, 17]. Propofol sedation may facilitate pharyngeal visualization by providing more stable sedation and suppressing gag reflexes during insertion. Similarly, previous investigator‐blinded studies have shown that propofol sedation may improve the technical quality of upper gastrointestinal endoscopy, particularly during endoscope insertion and passage through the pharyngeal region [10]. Based on previous randomized studies evaluating pharyngeal anesthesia during transoral endoscopy under midazolam sedation, inadequate suppression of the gag reflex negatively affected pharyngeal observation quality. Moreover, deeper sedation attenuated these differences [12, 17]. Collectively, these findings support the hypothesis that stable and sufficiently deep sedation may facilitate careful pharyngeal inspection during EGD.

The shorter procedure time observed with propofol should be interpreted cautiously because procedure duration is influenced by multiple procedural factors. Accordingly, procedure time was considered an exploratory measure and not a surrogate for examination quality.

Our findings suggest that sedation strategy may influence the technical quality and consistency of endoscopic examination during routine EGD. Previous studies have suggested that pethidine‐based conscious sedation may facilitate pharyngeal observation by suppressing the gag reflex while preserving patient cooperation with vocalization or the Valsalva maneuver [18]. In contrast, these maneuvers were not used in the present study. Therefore, the optimal sedation approach may differ depending on whether active patient cooperation is required for visualization of specific hypopharyngeal regions. Propofol may facilitate pharyngeal observation by providing rapid and relatively stable sedation, suppressing gag and swallowing reflexes, and reducing patient movement during endoscope insertion. These effects may allow the endoscopist to maintain a more stable view of the pharyngeal and hypopharyngeal mucosa. Although the reasons for unsuccessful pharyngeal observation were not systematically recorded in this retrospective study, review of the recorded images suggested that many unsuccessful cases were characterized by unstable visualization associated with pharyngeal movement during image acquisition, which may have reflected gagging or swallowing. Calendar period, included as a surrogate for temporal learning effects, was not independently associated with successful pharyngeal observation.

This study has several limitations. First, it was a retrospective single‐center observational study, and residual confounding could not be completely excluded despite multivariate adjustment. Second, the sedation strategy was not randomized. However, it was determined according to staffing availability and institutional operational conditions. This finding reflects real‐world clinical practice in regional training hospitals. Third, sedation depth was assessed clinically, and formal MOAA/S scores were not prospectively recorded. Therefore, differences in the achieved depth of sedation between the two groups cannot be excluded, and the observed association with successful pharyngeal observation cannot be attributed solely to the pharmacological properties of propofol. Future prospective studies using predefined sedation protocols and validated sedation scales, such as the MOAA/S, are warranted to distinguish the effects of the sedative agent from those of sedation depth. In addition, propofol is not routinely reimbursed for diagnostic EGD in Japan, and its availability varies among institutions, which may limit the generalizability of our findings. Fourth, the number of expert‐performed procedures was smaller than that of trainee‐performed procedures, which might have limited the statistical power of the subgroup analyses. Fifth, individual differences among trainees might have existed because the trainees originated from different institutions and had different sedation experiences. Nevertheless, operator experience and temporal learning effects were adjusted for in the multivariate analyses, and additional trainee‐only sensitivity analyses had consistent findings. Finally, calendar period was used as a surrogate marker of learning progression and might not completely capture the complexity of procedural learning curves.

In conclusion, sedation strategy was associated with successful pharyngeal observation during routine EGD. Propofol sedation may facilitate a more stable visualization during the insertion phase and support consistent pharyngeal inspection across operators with varying levels of experience.

## Author Contributions

**Conception and design**: Kazuya Sumi. **Data collection and treatment of patients**: Kazuya Sumi, Tomoya Sato, Tomona Sakurai, Kota Uehara, and Akira Ishihara. **Data analysis and interpretation**: Kazuya Sumi. **Drafting of the article**: Kazuya Sumi and Takayoshi Ito. **Critical revision of the article for important intellectual content**: Kazuya Sumi, Tomoya Sato, Tomona Sakurai, Kota Uehara, Akira Ishihara, and Takayoshi Ito. **Supervision**: Takayoshi Ito. All authors have approved the final version of the manuscript.

## Funding

The authors have nothing to report.

## Ethics Statement

**Approval of the research protocol by an Institutional Reviewer Board**: This retrospective study was approved by the Institutional Review Board of Hitachi Medical Center Hospital (approval number: 251111‐1) and conducted in accordance with the Declaration of Helsinki.

## Consent

The requirement for informed consent was waived due to the retrospective design of the study.

## Conflicts of Interest

The authors declare no conflicts of interest.

## Supporting information



Supporting File1: deo270422‐sup‐0001‐SuppMat.docx

## Data Availability

The datasets generated and/or analyzed during the current study are not publicly available due to patient privacy restrictions. However, they are available from the corresponding author upon reasonable request.
