# Supplementary material for: Association of Propofol Versus Midazolam Sedation With Successful Pharyngeal Observation During Upper Gastrointestinal Endoscopy: A Retrospective Study
Source: DEN Open. 2026 Sep 3;7(1):e70422. doi: 10.1002/deo2.70422 (PMC13540638; doi:10.1002/deo2.70422)
Supplement: Supplementary file 1 [file DEO2-7-e70422-s001.docx]

**Supplementary Material**

Supplementary Table 1. Multivariate logistic regression analysis excluding biopsy cases (n = 377)

| Variables | Odds ratio | 95% CI | P-value |
| --- | --- | --- | --- |
| Period | - | - | 0.668 |
| ASA-PS (III vs. I–II) | 0.33 | 0.07–1.57 | 0.183 |
| Operator (trainee vs. expert) | 0.39 | 0.17–0.87 | 0.013 |
| Indication (f/u vs. screening) | 1.16 | 0.54–2.49 | 0.700 |
| Propofol sedation | 2.46 | 1.29–4.68 | 0.005 |
| Age | 1.02 | 1.00–1.04 | 0.073 |
| Sex (M vs. F) | 0.85 | 0.47–1.55 | 0.600 |
| BMI | 0.97 | 0.91–1.05 | 0.481 |

f/u: follow-up

Supplementary Table 2. Multivariate logistic regression analysis among trainees (n = 327)

| Variables | Odds ratio | 95% CI | P-value |
| --- | --- | --- | --- |
| Period | - | - | 0.309 |
| ASA-PS (III vs. I–II) | 0.27 | 0.05–1.43 | 0.138 |
| Indication (f/u vs. screening) | 1.19 | 0.56–2.55 | 0.647 |
| Propofol sedation | 2.39 | 1.23–4.63 | 0.008 |
| Biopsy | 0.86 | 0.41–1.84 | 0.708 |
| Age | 1.03 | 1.01–1.04 | 0.006 |
| Sex (M vs. F) | 0.86 | 0.47–1.55 | 0.609 |
| BMI | 0.96 | 0.90–1.03 | 0.259 |

f/u: follow-up

Supplementary Table 3. Characteristics of the patients according to sedation agent after matching (n = 332)

|  | Before matching Standardized Difference | Propofol group (n = 166) | Midazolam group (n = 166) | P-value | After matching Standardized Difference |
| --- | --- | --- | --- | --- | --- |
| Age, years (mean ± SD) | 0.152 | 66.3 ± 15.4 | 66.0 ± 15.8 | 0.847 | 0.021 |
| Sex, male (%) | 0.102 | 50.0% (83/166) | 47.6% (79/166) | 0.742 |  |
| BMI, kg/㎡ (mean ± SD) | 0.049 | 23.3 ± 4.2 | 23.4 ± 4.0 | 0.879 | 0.017 |
| ASA-PS Ⅰ, Ⅱ | 0.034 | 161 (97.0%) | 162 (97.6%) | 1.000 | 0.037 |
| ASA-PS Ⅲ |  | 5 (3.0%) | 4 (2.4%) |  |  |
| Respiratory disease (%) | 0.082 | 8.4% (14/166) | 10.2% (17/166) | 0.707 | 0.062 |
| Cardiovascular disease (%) | 0.032 | 14.5% (24/166) | 13.9% (23/166) | 1.000 | 0.017 |
| Liver disease (%) | 0.064 | 3.6% (6/166) | 2.4% (4/166) | 0.750 | 0.070 |
| Renal disease (%) | 0.108 | 0.6% (1/166) | 1.2% (2/166) | 1.000 | 0.064 |
| Intracranial disease (%) | 0.009 | 5.4% (9/166) | 4.8% (8/166) | 1.000 | 0.027 |
| Psychiatric disorder (%) | 0.127 | 2.4% (4/166) | 5.4% (9/166) | 0.257 | 0.155 |
| Use of antithrombotic agents (%) | 0.123 | 17.5% (29/166) | 19.9% (33/166) | 0.673 | 0.062 |
| First-time endoscopy (%) | 0.045 | 19.3% (32/166) | 23.5% (39/166) | 0.422 | 0.103 |
| Screening (%) | 0.154 | 75.9% (126/166) | 76.5% (127/166) | 1.000 | 0.014 |
| Trainee (%) | 0.156 | 70.48% (117/166) | 70.48% (117/166) | 1.000 | 0.000 |
| Period |  |  | | 0.701 |  |
| Period 1 | 0.125 | 15.1% (25/166) | 12.6% (21/166) |  | 0.070 |
| Period 2 | 0.077 | 36.1% (60/166) | 41.0% (68/166) |  | 0.099 |
| Period 3 | 0.255 | 22.3% (37/166) | 18.7% (21/166) |  | 0.090 |
| Period 4 | 0.429 | 26.5% (44/166) | 27.7% (46/166) |  | 0.027 |
| Mean dose, mg (mean ± SD) |  | 63.5 ± 22.8 | 3.6 ± 0.9 |  |  |

BMI: body mass index; ASA-PS: American Society of Anesthesiologists physical status.

Supplementary Table 4. Comparison of endoscopic outcomes between sedation types in all procedures after matching (n = 332)

|  | Propofol group (n = 166) | Midazolam group (n = 166) | P-value |
| --- | --- | --- | --- |
| Successful pharyngeal observation (%) | 90.1 % (150/166) | 80.7% (134/166) | 0.0136 |
| Procedure time, min (mean ± SD) | 6.8 ± 2.6 | 7.5 ± 3.1 | 0.0174 |
| Biopsy performed (%) | 12.1% (20/166) | 17.5% (29/166) | 0.1599 |
| Hypotension (%) | 11.5% (19/166) | 13.9% (23/166) | 0.5164 |
| Oxygen desaturation (%) | 13.3% (22/166) | 11.5% (19/166) | 0.6121 |
| Patient satisfaction (0–10) | 9.5 ± 1.4 | 9.3 ± 1.8 | 0.3468 |
| Patient discomfort | 0.6 ± 1.8 | 0.8 ± 1.9 | 0.5750 |

VAS: Visual Analog Scale

Supplementary Table 5. Multivariate linear regression analysis for procedure time (n = 444)

| Variables | β coefficient | 95% CI | P-value |
| --- | --- | --- | --- |
| Period P1 (reference = P4) | 0.667 | 0.249–1.085 | 0.0018 |
| Period P2 (reference = P4) | 0.005 | −0.321 to 0.331 | 0.975 |
| Period P3 (reference = P4) | −0.153 | −0.513 to 0.206 | 0.403 |
| ASA-PS (III vs. I–II) | −0.184 | −0.865 to 0.497 | 0.595 |
| Operator (trainee vs. instructor) | 1.091 | 0.860 to 1.321 | <0.0001 |
| Indication (f/u vs. screening) | 0.024 | −0.221 to 0.270 | 0.846 |
| Propofol use | −0.348 | −0.558 to −0.139 | 0.0012 |
| Biopsy | 1.926 | 1.642–2.209 | <0.0001 |

f/u: follow-up
